# Developing a nomogram for predicting surgical intervention in pediatric intussusception after hydrostatic reduction

**DOI:** 10.3389/fped.2023.1092548

**Published:** 2023-05-31

**Authors:** Yize Zhuang, Xun Wang, Xia Fan, Fei Li, Guoqing He, Miao Luo, Yingming Tang

**Affiliations:** Department of Pediatric Surgery, Guizhou Provincial People’s Hospital, Guizhou, China

**Keywords:** intussusception, hydrostatic reduction, predictors, nomogram, risk factors

## Abstract

**Purpose:**

The aim of this study was to develop and validate a nomogram for predicting surgical intervention in pediatric intussusception after hydrostatic reduction.

**Methods:**

Children with intussusception who had treated with sonographically guided saline hydrostatic reduction as an initial treatment were enrolled in this study. The enrolled patients were randomly selected for training and validation sets, and the split ratio was 7:3. The medical records of enrolled patients were retrospectively reviewed. The patients were divided into a surgery and a non-surgery group according to the results of the nonsurgical reduction. A model for predicting the risk of surgical treatment was virtualized by the nomogram using logistic regression analysis.

**Results:**

The training set consisted of 139 patients and the validation set included 74. After logistic regression analysis using training set, duration of symptoms, bloody stools, white blood cells (WBCs), creatine kinase isoenzyme (CK-MB), long-axis diameter, poor prognostic signs by ultrasound and mental state were identified as the independent predictors of surgical intervention for intussusception. A model that incorporated the above independent predictors was developed and presented as a nomogram. The C-index of the nomogram in the validation set was 0.948 (95% CI, 0.888–1.000). The calibration curve demonstrated a good agreement between prediction and observation. The decision curve analysis (DCA) curve showed that the model achieved a net benefit across all threshold probabilities.

**Conclusion:**

Based on the predictors of duration of symptoms, bloody stools, WBCs, CK-MB, long-axis diameter, poor prognostic signs by ultrasound and mental state, we developed a nomogram for predicting surgical intervention after hydrostatic reduction. This nomogram could be applied directly to facilitate pre-surgery decision for pediatric intussusception.

## Introduction

Intussusception is the most common abdominal emergency in infants and toddlers, which is defined as the invagination of proximal bowel into the distal bowel (1, 2). The prevalence of intussusception is about 1–4 per 2000 infants and children, and the most common type is ileocolic intussusception (3). Early treatment is critical for intussusception in order to reduce the risk of secondary peritonitis, sepsis and bowel perforation (4).

Treatment modalities for intussusception include both non-surgery and surgery procedures. Pneumatic or hydrostatic reduction through anorectal enema is the main treatment of choice for the non-surgery procedure (5). Pneumatic reduction using air is very effective for intussusception treatment with advantages such as low cost and decreased risk of perforations (2). However, the use of the pneumatic reduction technique will increase the risk of exposure to radiation (6). Hydrostatic reduction using normal saline needs to be guided by ultrasound, which can avoid the radiation. Both of the 2 non-surgery techniques can be performed safely and exhibits high success rates (6). Currently, ultrasound-guided hydrostatic reduction has been suggested as the first choice for intussusception treatment in several studies, because it has a higher success rate, shorter hospital stay and no radiation risk comparing to pneumatic reduction (6–8). When non-surgery treatment is contraindicated or has failed, surgery procedures will be conducted.

Previously, risk factors for non-surgical intussusception reduction failure or predictors for surgery requirements have been investigated in several studies. For example, Khorana et al. (9) found that risk factors such as age, symptom duration, rectal bleeding and abdominal distension were closely associated with nonsurgical reduction failure. Wu et al. (10) identified that symptoms for at least 2 days before surgery and long intussusception were predictors for patients with intussusception at high risk of needing surgery. In addition, He and colleagues (11) found that sonographic features including the presence of peritoneal fluid and trapped fluid in the intussusception were the important risk factors for failure of hydrostatic intussusception reduction.

Although, the above works have contributed to the prediction of hydrostatic reduction failure, there is still lack of validated predictive tools. The aim of this study was to develop and validate a nomogram for predicting surgical intervention in pediatric intussusception after hydrostatic reduction.

## Materials and methods

### Patients

This retrospective study was approved by the Ethics Committee of Guizhou Provincial People's Hospital in accordance with the Declaration of Helsinki. Due to the nature of retrospective study, the informed consent was waived.

Inclusion criteria included the following: (1) all the patients enrolled were children below 7 years of age; (2) patients had a diagnosis of intussusception by a senior sonographer; (3) patients had treated with sonographically guided saline hydrostatic reduction as an initial treatment. Recurrent patients or patients with insufficient medical records were excluded from this study.

### Data collection

The enrolled patients were randomly selected for training and validation sets, and the split ratio was 7:3. The training set was used to train the prediction model. The validation data were used to validate the model.

The medical records of enrolled patients were retrospectively reviewed. All the enrolled patients were treated with hydrostatic reduction as an initial treatment. If non-surgery treatment was failure, surgery procedures would be conducted. The patients were divided into a surgery and a non-surgery group according to the results of the nonsurgical reduction.

The data collected included clinical features (such as, age, gender, weight, temperature at admission, vomiting, bloody stools, abdominal mass, mental status, and duration of symptoms), ultrasonic image characteristics (such as long-axis diameter and location of the mass, enlarged lymph node, absence of blood flow in the intussusception, and fluid trapped within the intussusception), and laboratory tests [white blood cells (WBCs), platelets (PLTs), C-reaction protein (CRP), fibrinogen (FIB), and creatine kinase isoenzyme (CK-MB), serum kalium (K), serum sodium (Na), and serum chloride (Cl)]. The sonographic poor prognostic signs of hydrostatic reduction were defined if one of the following signs was presented: enlarged lymph node in intussusception, thick peripheral hypoechoic rim, fluid trapped within the intussusception, free intraperitoneum fluid and the absence of blood flow in the intussusception. When the child had somnolence, listlessness, no crying and pale face, it indicated that the child's mental state was poor.

### Statistical analysis

The data in this study were analyzed by R4.0.3 software. There was no missing data for the final statistical analysis. On the basis of Harrell's guidelines (12), when the outcome is binary, the minimum value of the frequencies of the two response levels should be greater than 10 times the number of predictors. Our study included 73 patients with surgical reduction and 126 patients without surgical reduction. Thus the limiting sample size is 73, and based on Harrell's guidelines, no more than 7 predictors can be accommodated. The normal distribution of continuous variables was evaluated by using the Kolmogorov-Smirnov test. Continuous data with normal distribution were expressed as mean ± SD and compared by Student's *t*-test; data with a non-normal distribution were expressed by median with range and compared by Mann–Whitney *U* test. The qualitative data were expressed as percentages and compared by Chi-square or Fisher's exact tests. To assess independent predictors of surgical treatment for intussusception, a twostep approach was used. In the first step, the univariable analysis was done. Then, variables selected based on clinical and statistical grounds were included in a multivariable logistic regression model. The odds ratios (ORs) for surgical treatment were calculated by logistic regression analysis. A model for predicting the risk of surgical treatment was then virtualized by the nomogram based on these independent variables using the training set. The validation set was used to confirm the newly established nomogram. The concordance index (c-index) was used to evaluate the predictive accuracy of the model. Sensitivity and specificity were evaluated by receiver operating characteristics curve (ROC). Furthermore, the clinical value of the model was evaluated by decision curve analysis (DCA). A *p*-value < 0.05 was considered as statistically significant.

## Results

### Clinical characteristics

From July 2020 to February 2022, 210 patients with intussusception who had been treated with sonographically guided saline hydrostatic reduction as an initial treatment were screened in this study. Among them, 3 patients with age above 7 years old and 8 patients with insufficient medical records were excluded. Finally, a total of 199 patients who met the inclusion criteria were enrolled in this study. 139 patients were assigned to training set, while 60 patients were assigned to validation set. Surgical reduction was performed in 35.97% of cases (50/139) in training set and 38.33% of cases (23/60) in validation set, respectively. There were no significant differences between the two sets in surgical reduction (*p* = 0.751) or clinical characteristics. The comparative clinical characteristics among the training and validation sets are shown in Table 1.

**Table 1:** Demographic and clinical characteristics children with intussusception.

Variables	Training set (*n* = 139)	Validation set (*n* = 60)	*p*-value
Surgical reduction, *n* (%)	50 (35.97)	23 (38.33)	0.751
Age, *n* (%)			0.459
<12 months	26 (18.71)	12 (20.00)	
12–36 months	71 (51.08)	35 (58.33)	
>36 months	42 (30.21)	13 (21.67)	
Gender, *n* (%)			0.291
Male	105 (75.54)	41 (68.33)	
Female	34 (24.46)	19 (31.67)	
Weight, kg, mean ± SD	12.94 ± 5.38	13.04 ± 4.52	0.898
Duration of symptoms, *n* (%)			0.501
≤24 h	79 (56.83)	31 (51.67)	
>24 h	60 (43.17)	29 (48.33)	
Vomiting, *n* (%)	80 (57.55)	31 (51.56)	0.443
Bloody stools, *n* (%)	33 (23.74)	19 (31.67)	0.243
Abdominal mass, *n* (%)	22 (15.83)	6 (10.00)	0.278
Temperature, *n* (%)			0.941
≤37.8°C	121 (87.05)	52 (86.67)	
>37.8°C	18 (12.95)	8 (13.33)	
WBCs, ×10^9^/L, median (range)	11.25 (3.61, 40.54)	10.33 (4.54, 29.46)	0.451
PLTs, ×10^9^/L, median (range)	365.00 (121.00, 950.00)	347.50 (152.00, 763.00)	0.857
CRP, mg/L, median (range)	6.1 (0.10, 100.63)	6.13 (0.26, 55.26)	0.853
FIB, g/L, median (range)	3.24 (1.50, 31.60)	3.21 (0.52, 22.45)	0.888
CK-MB, U/L, median (range)	35.40 (15.70, 126.00)	36.35 (14.00, 124.00)	0.850
Long-axis diameter (Ultrasound)			0.950
≤4 cm	106 (76.26)	46 (76.67)	
>4 cm	33 (23.74)	14 (23.33)	
Poor prognostic signs by ultrasound	75 (53.96)	29 (48.33)	0.466
Enlarged lymph node (> 1 cm)	66 (47.48)	23 (38.33)	0.234
Absence of blood flow in the intussusception, *n* (%)	10 (5.92)	5 (8.33)	0.780
Fluid trapped within the intussusception, *n* (%)	26 (18.71)	15 (25.00)	0.314
Location, *n* (%)			0.428
Right side	99 (71.22)	46 (76.67)	
Left side	40 (28.78)	14 (23.33)	
Mental state, *n* (%)			0.760
Good	82 (58.99)	34 (56.67)	
Poor	57 (41.01)	26 (43.33)	
K, mmol/L, mean ± SD	4.48 ± 0.65	4.48 ± 0.59	0.945
Na, mmol/L, mean ± SD	136.38 ± 3.80	136.82 ± 4.29	0.655
Cl, mmol/L, mean ± SD	106.11 ± 4.37	105.85 ± 4.35	0.710

WBCs, white blood cells; PLTs, platelets; CRP, C-reaction protein; FIB, fibrinogen; CK-MB, creatine kinase isoenzyme; K, kalium; Na, Sodium; Cl, Chloride.

### Development of the nomogram

**Figure 1:**
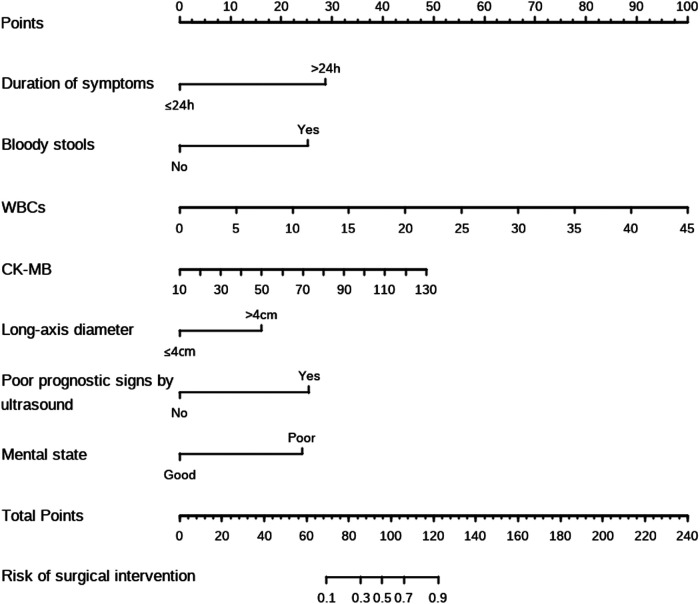
The nomograms for predicting the risk of surgical intervention in pediatric intussusception after hydrostatic reduction.

The univariable analysis using the training set showed that duration of symptoms (*p* < 0.001), vomiting (*p* = 0.001), bloody stools (*p* < 0.001), abdominal mass (*p* < 0.001), temperature (*p* = 0.002), WBCs (*p* < 0.001), PLTs (*p* < 0.001), CRP (*p* = 0.006), CK-MB (*p* = 0.041), long-axis diameter (*p* = 0.004), poor prognostic signs by ultrasound (*p* < 0.001), absence of blood flow in the intussusception (*p* = 0.006), serum Na (*p* < 0.001), mental state (*p* < 0.001) and intussusception location (*p* < 0.001) were the significant factors for surgical intervention of intussusception. These 15 clinical candidate predictors were selected for the multivariable analysis. After multivariable analysis was done, duration of symptoms (odds ratio [OR] = 10.901; 95% confidence interval [CI] = 3.131–37.957; *p* < 0.001), bloody stools (OR = 8.202; 95% CI = 2.168–31.029; *p* = 0.002), WBCs (OR = 1.203; 95% CI = 1.023–1.416; *p* = 0.026), CK-MB (OR = 1.034; 95% CI = 1.002–1.068; *p* = 0.040), long-axis diameter (OR = 3.809; 95% CI = 1.006–14.429; *p* = 0.049), poor prognostic signs by ultrasound (OR = 8.303; 95% CI = 2.288–30.132; *p* = 0.001), and mental state (OR = 7.426; 95% CI = 2.217–24.872; *p* = 0.001) were identified as the independent predictors of surgical intervention for intussusception. The results of the univariable and multivariable analyses are listed in Table 2. Then, a model that incorporated the above independent predictors was developed and presented as a nomogram (Figure 1).

**Table 2:** Univariable and multivariable analyses of the predictors of surgical intervention.

Variables	Univariable analysis		Multivariable analysis	
	OR [95% CI]	*p*-value	OR [95% CI]	*p*-value
Age	1.239 [0.747–2.056]	0.407	–	–
Gender				
Male	Reference		–	–
Female	0.962 [0.429–2.158]	0.925	–	–
Weight	0.922 [0.848–1.002]	0.057	–	–
Duration of symptoms				
≤24 h	Reference		Reference	
>24 h	0.104 [0.046–0.233]	<0.001	10.901 [3.131–37.957]	<0.001
Vomiting	3.544 [1.639–7.661]	0.001	–	–
Bloody stools	12.690 [4.906–32.830]	<0.001	8.202 [2.168–31.029]	0.002
Abdominal mass	11.953 [3.758–38.022]	<0.001	–	–
Temperature				
≤37.8°C	Reference		–	–
>37.8°C	5.903 [1.962–17.760]	0.002	–	–
WBCs	1.187 [1.084–1.299]	<0.001	1.203 [1.023–1.416]	0.026
PLTs	1.007 [1.004–1.011]	<0.001	–	–
CRP	1.033 [1.009–1.056]	0.006	–	–
FIB	0.987 [0.898–1.085]	0.784	–	–
CK-MB	1.020 [1.001–1.039]	0.041	1.034 [1.002–1.068]	0.040
Long-axis diameter				
≤4 cm	Reference		Reference	
>4 cm	3.283 [1.465–7.361]	0.004	3.809 [1.006–14.429]	0.049
Poor prognostic signs by US	6.171 [2.737–13.915]	<0.001	8.303 [2.288–30.132]	0.001
Enlarged lymph node	1.505 [0.750–3.019]	0.250	–	–
Absence of blood flow	19.317 [2.368–157.583]	0.006	–	–
Fluid trapped within the intussusception	2.054 [0.866–4.870]	0.102	–	–
K	0.653 [0.374–1.139]	0.653	–	–
Na	0.819 [0.733–0.915]	<0.001	–	–
Cl	0.919 [0.839–1.007]	0.072	–	–
Mental state				
Good	Reference		Reference	
Poor	8.327 [3.788–18.305]	<0.001	7.426 [2.217–24.872]	0.001
Location				
Right side	Reference		–	–
Left side	3.294 [1.942–5.586]	<0.001	–	–

WBCs, white blood cells; PLTs, platelets; CRP, C-reaction protein; FIB, fibrinogen; CK-MB, creatine kinase isoenzyme; K, kalium; Na, Sodium; Cl, Chloride; OR, Odds ratio; CI, confidence interval; US, ultrasound.

### Validation of the nomogram

**Figure 2:**
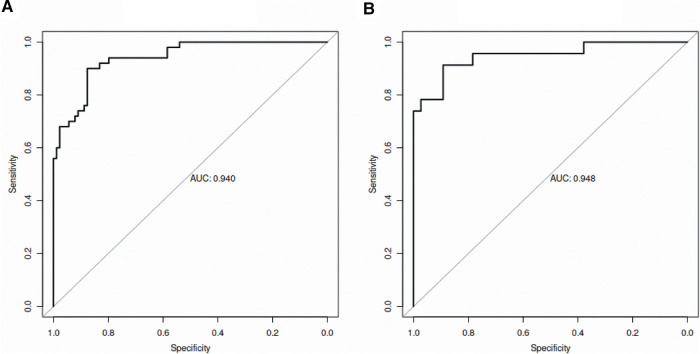
ROC curves of the established model in the training set (**A**) and validation set (**B**). ROC, receiver operating characteristic; AUC, area under the curve.

The C-index for the prediction nomogram was 0.940 (95% CI, 0.903–0.978) in the training set (Figure 2A). The Hosmer-Lemeshow test found no statistical significance in the training set (*p* = 0.174).

**Figure 3:**
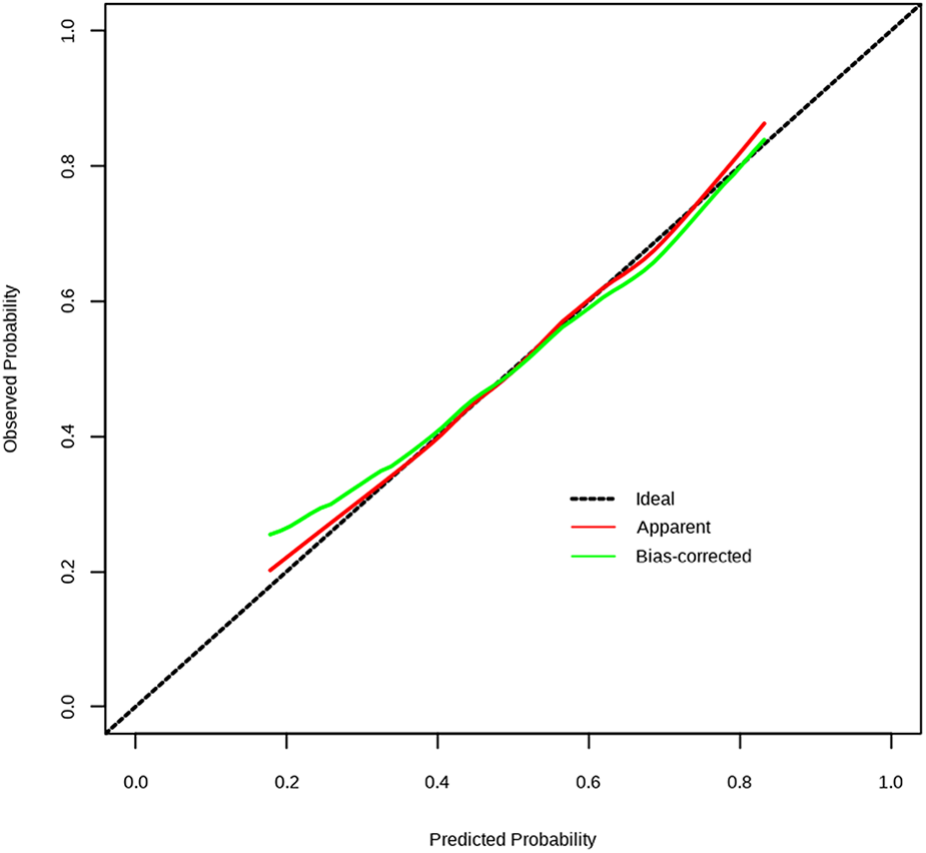
Calibration curve of the model in the validation set. The Y-axis represents the actual surgical intervention rate. The x-axis represents the predicted risk of surgical intervention. The dotted line represents a perfect prediction by an ideal model. The green line (bias corrected) represents the bootstrap-corrected performance of our nomogram, and the red line (apparent) represents the apparent accuracy of the nomogram.

**Figure 4:**
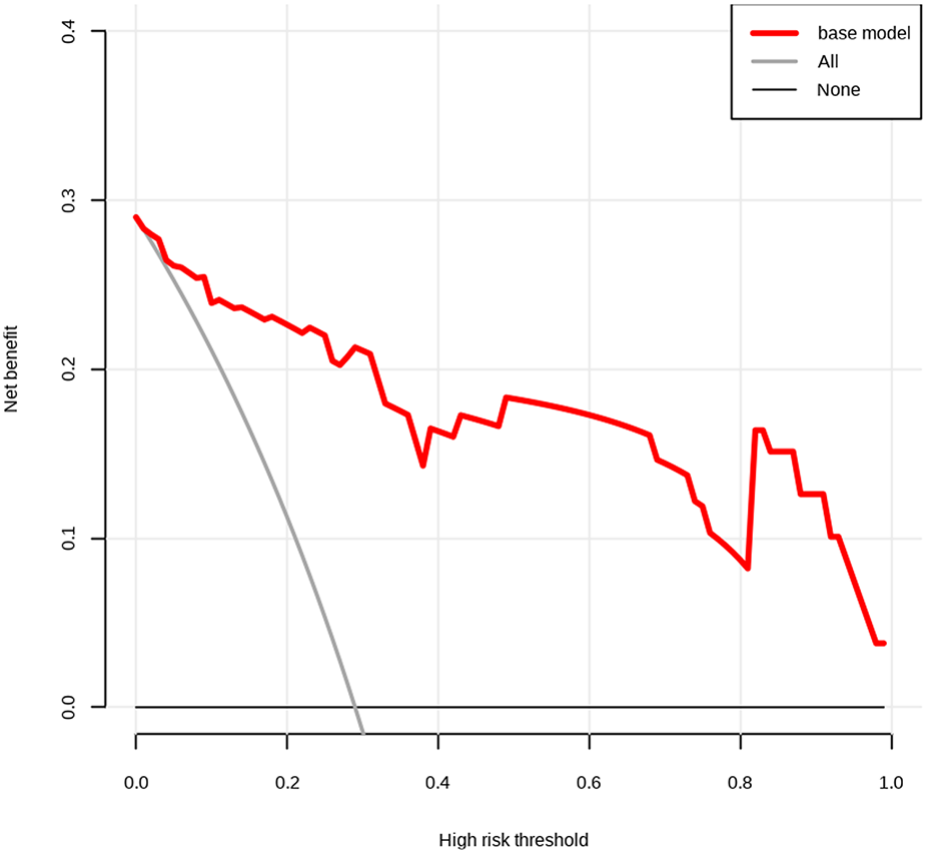
Decision curve analysis (DCA) for the predictive model. The net benefit was produced against the high-risk threshold. The red line represents the predictive model. The application of this predictive model would add net benefit compared with either the treat-all or the treat-none strategies.

The C-index of the nomogram for the prediction of surgical intervention for intussusception in the validation set was 0.948 (95% CI, 0.888–1.000, Figure 2B). The calibration curve demonstrated a good agreement between prediction and observation in the validation set (Figure 3). Furthermore, the Hosmer-Lemeshow test yielded a nonsignificant statistic in the validation set (*p* = 0.873), indicating that no departure from a perfect fit was found. To evaluate the clinical usefulness of the model, we conducted DCA in the validation set. The DCA curve showed that the model achieved a net benefit across all threshold probabilities (Figure 4).

## Discussion

In the present study, an effective nomogram that used clinicopathologic risk factors to predict surgical intervention in pediatric intussusception was developed and validated. Risk factors such as duration of symptoms, bloody stools, WBCs, CK-MB, long-axis diameter, poor prognostic signs by ultrasound and mental state were incorporated into the nomogram based on the training set. In the validation set, our nomogram was identified as a highly predictive tool that could be applied directly.

Nomograms are a pictorial representation of a complex mathematical formula (13). To our best knowledge, there are few studies focusing on the development of nomograms for intussusceptions. Recently, Ting et al. (14) developed a nomogram to predict pathological intussusceptions in children. Episode time, mass length and infection history were identified as the independent predictors (14). In the present study, we used multiple logistic regression analysis to develop a preoperative nomogram that predicted surgical intervention for pediatric intussusception after hydrostatic reduction. Duration of symptoms, bloody stools, WBCs, CK-MB, long-axis diameter, poor prognostic signs by ultrasound and mental state were regarded as the predictors incorporating in the nomogram.

It has been reported that long duration of symptoms of intussusceptions was related to the increase of intestinal viability loss (9). Thus, several previous studies indicated that duration of symptoms was associated with failed non-surgery reduction. For instance, Reijnen et al. (15) found that a duration of symptoms >48 h was a risk factor for failure of hydrostatic reduction. Khorana et al. (9) reported that duration of symptoms >72 h was one of the predictors of failed non-surgery reduction (9). Similarly, Huang et al. (16) demonstrated that duration of symptoms was the independent risk factor for surgical reduction of intussusceptions. In this study, we also found that duration of symptoms was an independent predictor for surgical intervention after hydrostatic reduction. However, the cutoff value of this study was set as 24 h which was different from the studies mentioned above. The reason may be due to the duration of symptoms in the most of enrolled patients in this study did not reach 48 h.

Bloody stools are one of the classic signs of intussusception, which have been regarded as the poor predictor of intussusception (17). In 2014, He et al. (11) retrospectively reviewed the medical records of 288 cases of intussusception and found that bloody stool is one of the most important risk factors for failure of hydrostatic intussusception reduction. Consistent with He's study, Khorana et al. (9) also identified that bloody stool was a predictor of failed reduction. As expected, using multiple logistic regression analysis, the presence of bloody stools was also demonstrated as an independent predictor for surgical intervention after hydrostatic reduction in the present study.

Our study demonstrated for the first time that WBCs count and CK-MB were independent predictors for surgical intervention of intussusception. Although limited studies have revealed the relationship between WBCs count and failure of hydrostatic intussusception reduction, the association between WBCs count and intestinal necrosis has been revealed previously. In Chen's study, they found that patients underwent intestinal resection due to intestinal necrosis had significantly higher WBCs count than those without any resection (18). In 2020, Chen et al. (19) developed a prediction model that used WBCs count as an important risk factor. Chen's prediction model has been validated to predict the occurrence of intestinal necrosis well (19). It has been reported that CK-MB in blood could reflect ischemia of intestinal smooth muscle to some extent (19). Intestinal ischemia will result in intestinal necrosis, causing the synthesis of large amounts of CK or CK-MB into the blood. A previous study showed that the level of CK-MB in blood was markedly evaluated in the rat model of intestinal ischemia (20). Thus, the CK-MB may also relate to the presence of intestinal necrosis. Since the intestinal necrosis is the main reason for non-surgical intussusception reduction failure or surgery requirements, we have reasons to believe that WBCs count and CK-MB maybe associated with surgical intervention after hydrostatic reduction. But this conclusion should be confirmed by further studies with larger sample size.

Long-axis diameter is one of the important factors affecting the success rate of non-surgical intussusception reduction. Duc et al. (21) found that surgical intussusception had a significantly smaller diameter than non-surgical intussusception. Zhang et al. (22) reported that intussusception recurrence was prone with greater mass diameter. Consistent with previous studies, logistic regression analysis used in this study showed that long-axis diameter was identified as an independent predictor of surgical intervention for intussusception. Longer diameter was prone with higher risk of surgical intervention.

Diarrhea and vomiting are early symptoms of intussusception (23). In this study, the children may have vomit and diarrhea before hospitalization, which may cause dehydration of the children, with listless, lethargic, pale and other symptoms. Poor mental state reflects dehydration of the child. Patients with dehydration showed significantly higher risk of failure in treatment using hydrostatic reduction (24). Therefore, mental state as a prognostic factor of intussusception was of great significance. The poor prognosis signs by ultrasound were reported to be associated with the reduction failure in many studies. Khorana et al. (25) found that the poor prognosis signs by ultrasound was an important prognostic factor for the nonsurgical reduction. Furthermore, poor prognosis signs by ultrasound were also included as a prognostic factor for failed reduction in Chiang Mai University Intussusception Failed Score (CMUI) (26). This scoring system showed a high specificity and a high affinity for prediction of failed reduction (26). In the present study, the poor prognosis signs by ultrasound were identified as an independent predictor for surgical intervention as found in previous studies.

Other risk factors such as age, light weight (9, 25), temperature >37.8°C (9, 22) and location of mass (9) were identified as independent predictors for non-surgical intussusception reduction failure in previous studies. However, in the present study, these factors did not show strong association with surgical intervention after hydrostatic reduction. The rejection of these factors in the nomogram may be the result of nuances in the data set or confounding by other predictors (27).

After the nomogram was constructed, we validated it in the validation set. The AUC of the model was as high as 0.907 in the validation set. Additionally, calibration curve analysis and Hosmer-Lemeshow test further showed good calibration of our model in the validation set. Finally, we evaluated the clinical usefulness of the model by DCA and found that the model achieved a net benefit across all threshold probabilities, indicating that the nomogram has high clinical usefulness in our studied population.

The present study has several limitations. First, the nomogram was established based on data obtained from a single center in China. This may cause selection bias. Second, all data in the present study were collected retrospectively and the sample size of this study was relatively small. Finally, the nomogram we developed did not be validated by external dataset. Further prospective studies are needed to validate our model.

## Conclusion

In conclusion, we developed a nomogram based on clinical risk factors to predict surgical intervention in pediatric intussusception after hydrostatic reduction. Duration of symptoms, bloody stools, WBCs, CK-MB, long-axis diameter, poor prognostic signs by ultrasound and mental state were identified as predictors and incorporated into the nomogram. Our internal validation demonstrated that this nomogram could be applied directly to facilitate pre-surgery decision for pediatric intussusception. However, our nomogram is just the adjunct for the pre-surgery decision and cannot be used instead of the contraindication for the non-surgical reduction.

## Data Availability

The original contributions presented in the study are included in the article, further inquiries can be directed to the corresponding author.
